# Advances in Electrically and Thermally Conductive Functional Nanocomposites Based on Carbon Nanotubes

**DOI:** 10.3390/polym17010071

**Published:** 2024-12-30

**Authors:** Alexandr V. Shchegolkov, Aleksei V. Shchegolkov, Vladimir V. Kaminskii, Pablo Iturralde, Maxim A. Chumak

**Affiliations:** 1 Institute of Power Engineering, Instrumentation and Radioelectronics, Tambov State Technical University, Tambov 392000, Russia; 2 Center for Project Activities, Advanced Engineering School of Electric Transport, Moscow Polytechnic University, Moscow 107023, Russia; alexxx5000@mail.ru; 3 Institute of Advanced Data Transfer Systems, ITMO University, St. Petersburg 197101, Russia; kam-vladimiro@yandex.ru; 4 Advanced Engineering School of Electric Transport, Moscow Polytechnic University, Moscow 107023, Russia; iturralde.p@gmail.com; 5 Centre of Nanoheterostructure Physics, Ioffe Institute, Saint Petersburg 194021, Russia; equilibrium2027@yandex.ru

**Keywords:** polymer, electrical conductivity, thermal conductivity, nanocomposite, carbon nanomaterials, functional materials, strain gauge, electric heater

## Abstract

The paper presents a review of CNTs synthesis methods and their application as a functional filler to obtain polymer composites for various technical purposes for strain gauges, electrical heating, anti-static coatings, electrically conductive compounds, etc. Various synthesis methods allow CNTs with different morphology and structural properties to be created, which expands the possibilities of the application of such nanoscale structures. Polymers can provide such effects as ‘shape memory’ and self-repair of mechanical defects. Different combinations of polymers and dispersed fillers influence the change in electrical and thermal conductivity, as well as the positive temperature coefficient of resistance, which makes it possible to achieve the effect of temperature self-regulation during electrical heating. CNTs make it possible to form PTCR (positive temperature coefficient of resistance) in elastomers at lower concentrations, which makes it possible to preserve mechanical strength and use more efficient modes of heat generation. For strain gauges, CNTs improve sensitivity to mechanical effects and extend the measurement range. The use of thermoplastic elastomers provides the temperature of PTCR operation for electric heating at the level of 200 °C (voltage 240 V), which allows such heaters to operate at a power supply from a household electrical network. CNTs-based strain gauges can provide structural condition monitoring of composite materials.

## 1. Introduction

Various electronic devices based on nanostructured carbon are considered promising and are alternatives to replace or supplement silicon-based electronic device components [1]. Functionalized polymer nanocomposites (FPNs) containing carbon nanotubes (CNTs) have a wide range of applications [2,3,4,5,6,7,8]. These materials can be used as sensors [4,5,6] and electric heating elements [7,8]. The thermal resistivity properties of CNTs and polymer nanocomposites based on them are becoming important [2]. The influence of tunneling and electron hopping on the temperature coefficient of co-protection of polymer nanocomposites with CNTs has been established [3]. This makes it possible to realize piezoresistive strain sensors from polymer nanocomposites based on CNTs [4] or a combination of CNTs, graphene, and nano-aluminum [5]. It is also worth noting the possibility of monitoring the state of composites and the realization of them becoming full-fledged using electrical resistivity tomography when using CNTs [6].

**Figure 1:**
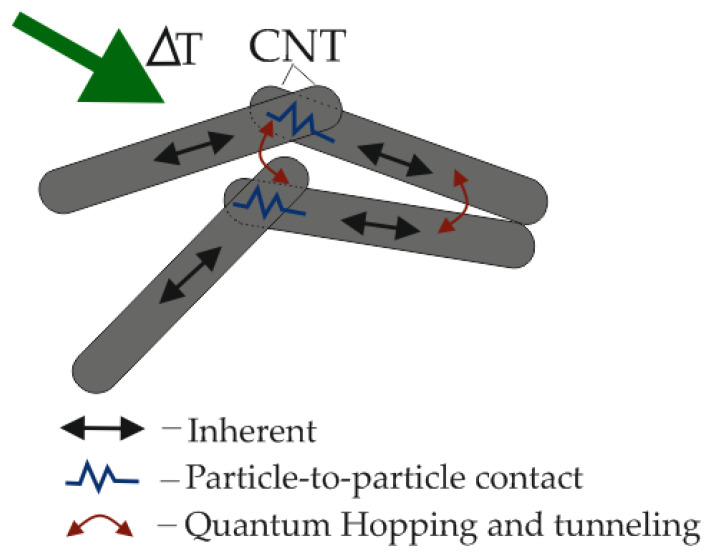
Thermoresistive mechanisms occurring in CNT arrays.

Polymer composites can be effective electric heaters with the effect of temperature self-regulation based on polyethylene [7] and silicon-organic compounds [8] with CNT-based filling. The wide application of CNTs leads to various methods of their synthesis [9]. FPNs have attracted the attention of a wide range of researchers due to their mass-size characteristics, and physicochemical, mechanical, anticorrosion, and other properties. As an example, thermoresistive mechanisms arising in CNT arrays are proposed [4,10]. Conventionally, they can be divided into three mechanisms (Figure 1):

- Inherent;
- Particle-to-particle contact;
- Quantum Hopping and tunneling.

Through engineering design methods and molecular modeling approaches [10], polymers can be modified with other polymers and copolymers as well as dispersed fillers, allowing them to be tailored for various applications [11].

A significant advantage of polymers over other types of materials is the possibility of creating self-healing composites [12]. At the same time, the memory effect is available for polymers [13]. Polymers are effectively combined with different types of carbon nanostructures, such as CNTs obtained by different methods [5]. At the same time, CNTs due to the formation of a synergistic effect, i.e., the combination of a flexible or highly elastic backbone (polymer matrix) and dispersed filler, are able to possess one or more functional properties. Such polymers have been called “smart” or intelligent polymers [14,15]. For example, FPNs can have temperature-dependent electrophysical characteristics, in which the effect of temperature self-regulation is realized [16,17]. Based on polymer composites, it is possible to create effective strain gauge transducers with high sensitivity and a wide range of strain (tension, compression, and torsion) and mechanical stress measurements [18].

The aim of the review study is to analyze the methods of synthesis of carbon nanotubes as filler in polymers to create FPNs for a wide range of applications. To achieve the goal, the following tasks were formulated and solved:

- Analysis of CNTs synthesis methods;
- Study of various polymers used as backbone in composite materials;
- Analysis of practical application of PFM for local heating.

## 2. Methods of CNT Synthesis

CNT synthesis can be realized by a variety of methods and technologies [5,19,20]. These methods are based on various physicochemical and mechanical approaches, which can be realized by means of laser technology, continuous or batch reactors, arc discharge process in gaseous and liquid media, microwave action on carbon-containing materials, etc.

**Figure 2:**
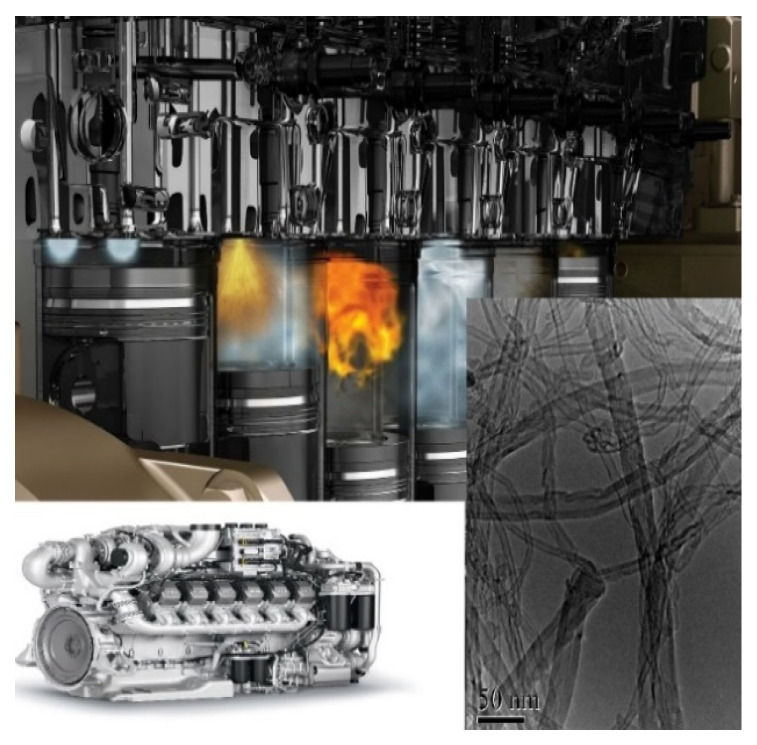
Installation for CNT synthesis [21].

The synthesis of CNTs is influenced by various factors such as pressure, temperature, and volume of the reaction zone where the synthesis takes place. In this respect, there is a synthesis technology using as a reactor a diesel engine with compression ignition [21], as presented in Figure 2. It follows from Figure 2 that the thermal processes in the combustion chamber provide the necessary conditions for CNT synthesis, which allow the nanomaterial to be produced in a constant mode of operation (Four-stroke operation of the engine leads to the fact that where the orange colour—is the process of combustion (second cylinder)—the stage of synthesis, the first cylinder the beginning of the combustion process and the third purging—exhaust gas release.). Ethanol-based biofuel can be used as a fuel. At certain modes of operation of an internal combustion engine from compression, solid particles are formed in the exhaust gases, which have a morphological structure similar to CNTs [22,23].

The growth mechanism and wave-absorbing properties of multilayer carbon nanotubes fabricated by gas detonation are presented in [24]. Gas detonation synthesis of Co@C/CNTs nanoparticles is presented in [25]. Further in the review, the most common methods of CNT synthesis are presented.

### 2.1. Arc Discharge Method

CNT synthesis using electric arc discharge is one of the common production methods, which is based on heating carbon material to a very high temperature in a confined environment (gaseous or aqueous), which subsequently leads to the formation of CNTs [26].

Ionic compounds of platinum group metals (PGMs): Ru, Rh, Pd, Os, Ir, and Pt can be used for the electric arc synthesis of CNTs [27]. This approach makes it possible to synthesize defect-free CNTs using PGMs as catalysts. The technology of galvanic deposition of the catalyst on the cathode can be used in the process of CNT synthesis under DC arc discharge [28].

In [29], high-purity graphite powder served as the carbon source and iron served as the catalyst for the synthesis of defect-free multi-walled carbon nanotubes (MWCNTs) by DC arc discharge in a nitrogen atmosphere. Fe has a high defect reduction efficiency, which promotes the synthesis of MWCNTs with an ideal lattice and without defects.

Selective growth of single-walled carbon nanotubes (SWCNTs) with a selected diameter (in the range of 1.5–1.7 nm) was carried out by DC arc discharge, in which carbon monoxide (CO) mixed with helium was used as buffer gases [30]. In [31], arc discharge in water was used to synthesize CNTs. Copper rods and graphite rods are used as cathodes and anodes in the synthesis of CNTs. In addition to the 50 A DC current, a perpendicular magnetic field is also applied to the electrodes during CNT synthesis.

Immediately after synthesis (constant electric field), SWCNTs can aggregate into bundles with diameters of several tens of nanometers, and these bundles are very difficult to separate after synthesis [32]. In [33], the synthesis of dahlia-like hybrids was shown along with thin bundles (average diameter ~ 5.7 nm) of SWCNTs with unique aggregates of nanorods of almost spherical shape and relatively smaller size (average size ~ 25 nm).

### 2.2. CVD Method

CVD (Chemical vapor deposition) is the most common and efficient method for synthesizing carbon nanotubes (CNTs) with high purity and crystallinity, which is based on chemical reactions occurring in the gas phase at high temperatures using a hydrocarbon starting material.

In the CVD process, it is possible to control the morphology, structure, and size of CNTs by adjusting the temperature, pressure, and composition of the gas mixture and the type of catalyst [34]. The formation of carbon particles on the catalyst surface, which have a decisive effect on particle growth leading to the cessation of CNT growth on the particles [35]. Temperature, pressure, and acetylene flow rate affect the mechanisms that limit catalyst deactivation and hence CNT growth during CVD synthesis [36]. In [37], the effect of the crystallinity of sapphire substrate on the growth rate, efficiency, quality, and structure of CVD-synthesized oriented carbon nanotubes (CNTs) was investigated.

In [38], the role of sulfur in the synthesis of CNTs by the CVD method is evaluated. The introduction of sulfur into the iron triad nanoparticle (with emphasis on Fe as the most common one) has a complex effect: the melting temperature decreases, the surface tension drops due to surface segregation, and the solubility of carbon decreases.

**Figure 3:**
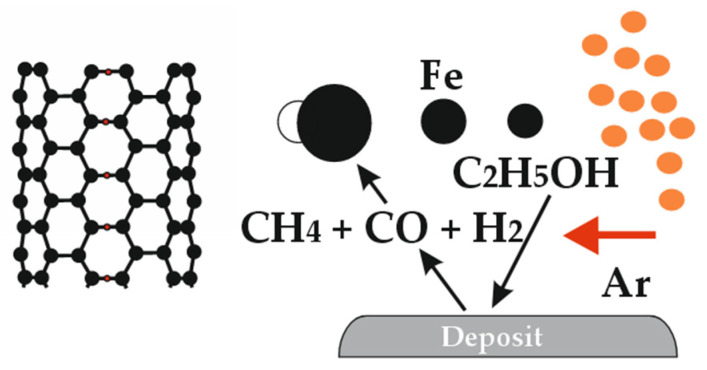
Mechanism of CNT formation.

In [39], the obtained SWCNTs demonstrate a stable distribution of chiral indices in a wide range of specified temperatures (750–1000 °C). At the same time, the carbon precipitate on the reactor wall plays an important role in maintaining the optimal mode of CNT synthesis (Figure 3) (orange colour shows drops of alcohol with dissolved ferrocene).

The effect of hydrogen added to the CO atmosphere as a growth stimulant on the synthesis and properties of SWCNTs was investigated in [40]. Optimization of floating catalyst CVD to maximize the yield and minimize the diameter of SWNTs, taking into account the complex dependence and interaction effects between the process parameters, is presented in [41].

In [42], a strategy for the optimal development of a multi-parameter CNT synthesis process is presented.

In [43], a mathematical model of the formation and growth of iron particles in a chemical reactor is described, which the authors used to study the formation of CNTs. Ferrocene decomposed with the formation of iron particles, which catalyzed the formation of CNTs from decomposed ethanol.

In [44], a catalytic chemical vapor deposition (CCVD) method was used to synthesize CNTs using Fe/CaCO_3_ and acetylene as a catalyst and a hydrocarbon source, respectively. The synthesis was carried out for (30–60 min) with reaction temperature (700–800 °C) and catalyst loading (10–30 wt.% Fe).

Plasma-enhanced chemical vapor (PECVD) synthesis of CNTs at controlled pressures (10 torr to 400 torr) forms CNTs with diameters ranging from 25 nm to 250 nm [45].

It is technically important to obtain VACNTs on non-planar surfaces and to synthesize VACNT hybrids with a core–shell structure. To achieve this goal, atomic layer deposition (ALD) is widely used [46].

### 2.3. Laser Ablation

CNT synthesis by laser ablation is a method that uses laser radiation to remove carbon matter from the surface of a material to form carbon nanotubes. Laser radiation is directed at the top layer of solid carbon material (graphite or amorphous carbon), which results in strong heating of this surface and subsequent rapid synthesis reaction. As a consequence, carbon nanoparticles are formed, which join together to form CNTs.

In [47], the preparation of MWCNTs and carbon nanoparticles by pulsed laser ablation of a graphite target in water without the use of a catalyst was demonstrated. The average diameter of CNTs synthesized at 532 nm was 20 nm and had a length of a few micrometers, whereas CNTs synthesized at 1064 nm had an average diameter of 75 nm and a length of a few submicrometers.

The influence of the laser wavelength on the yield of SWCNT synthesis and their properties was studied in [47]. Under UV radiation, the properties of synthesized CNTs depend on the laser parameter to a much greater extent than in the case of infrared laser radiation. Direct laser vaporization of transition metal/graphite composite rods obtained CNTs in condensing vapor in a heated flow tube [48]. A mixture of Co and Ni catalyzed about 50% of the total carbon evaporated to SWCNTs [49]. A method was developed to produce CNTs (high degree of graphitization with an inner diameter of 3–10 nm and an outer diameter of 10–100 nm) using catalytic decomposition of acetylene in a mixture with argon, by laser evaporation of solid nickel in situ [50].

In [51], the synthesis of MWCNTs in a 1473 K furnace using a copper vapor laser (CVL) is reported. The operating parameters of this laser, i.e., high energy density at the focus and high frequency of 10 kHz, distinguished it from conventional laser sources for CNT synthesis.

The results of [52]’s experimental study of the regularities of synthesis of single-walled carbon nanotubes (SWCNTs) by laser ablation of a graphite target with a catalyst and continuous CO_2_ laser radiation are presented. The rapid synthesis of Single-Walled Carbon Nanotube/Copper was performed using a laser (455 nm, xTool D1 Pro, xTool) at 1–8 W power [52].

### 2.4. Microwave Radiation

Microwave irradiation can be used for carbon graphitization and production of graphene oxide (GO) using salts of various metals (Ni, Co, Fe, Cr) as catalysts [53]. Carbon materials can differ significantly in the number and quality of layers, the level of doping, and confinement [54].

The strategy of microwave-assisted carbon nanofiber (CNF) growth on the “self-assembled” carbon multi-point structure (CU) maintains the hierarchy level, which improves electrical contact. To obtain the 3D structure, the classical ionomer pair, PEDOT:PSS, was chosen as a template [55]. By selecting the substrate and catalyst, low-temperature CNT growth can also be achieved by microwave synthesis on organic polymers with a low melting point and atmospheric pressure [56].

Ultrafast growth of carbon nanotubes using microwave irradiation is presented in [22]. Microwave technology can affect the properties of CNTs, which leads to the reduction in agglomerates in polymer matrices and improvement in their distribution [57]. The rapid growth of CNTs is associated with the aggregated morphology and subsequent transformation process of iron nanoparticles [58]. A simple synthesis of carbon nanotubes by low-temperature pyrolysis of ferrocene is presented in [59]. The synthesis of CNTs by pyrolysis of ferrocene and glycerol is shown in [60].

Microwave technology allows synthesizing heterostructures in which CNTs will be on the graphite surface [61]. The authors [62] used microwave heating of WO_3_, Co_3_O_4_, and C_3_N_3_ (NH_2_)_3_ powders, which allowed obtaining CNTs/WC for 10 min (900 °C). CNTs/WC are not prone to CNTs agglomeration and are uniformly dispersed. The microwave curing method for obtaining nanocomposites with a CNT content of 0.5 to 2.5 wt.% allowed improving electrical conductivity [63]. The type of substrate on which CNTs are synthesized affects the emissivity. The emissivity of CNTs on a silicon substrate is higher than on a quartz substrate [64].

### 2.5. Functionalization of CNTs

**Figure 4:**
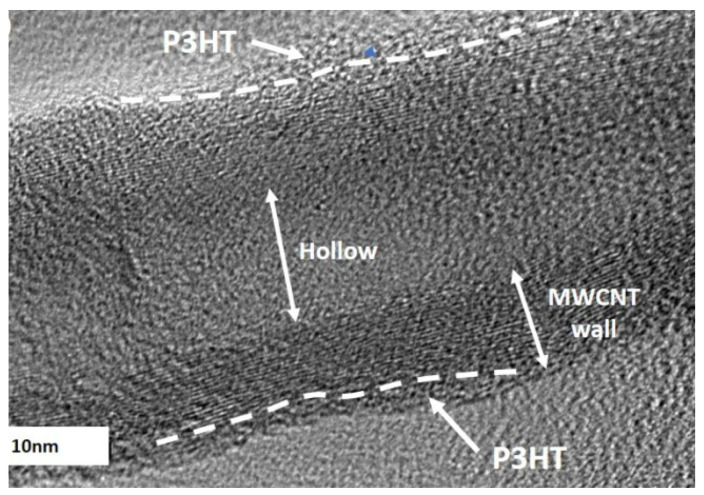
HRTEM image of P_3_HT/MWCNT-OH nanocomposites [68].

The use of native CNTs is difficult due to poor interaction with polymer matrices. The interaction characteristics of CNTs can be significantly improved by using the technological technique of functionalization [65,66]. Surface functionalization by chemical treatment allows their bundles to unravel, allowing dissolution and stabilization in solvents. Methods for covalent and non-covalent functionalization of aligned and unaligned CNTs with various heteroatomic dopants, functional groups, small molecules, and/or macromolecules have been developed [67]. Non-covalent functionalization of carbon nanotube sidewall via polymer wrapping is a key strategy to improve well-dispersed CNTs (Figure 4) [68].

**Figure 5:**
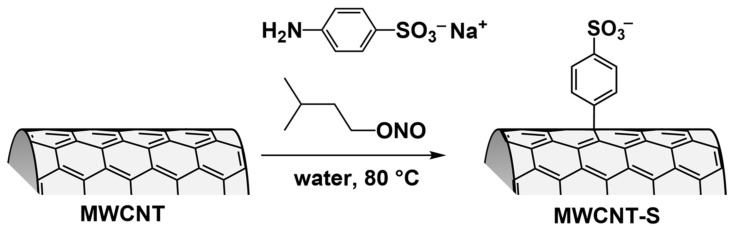
Synthesis of MWCNT-PhSO_3_—(MWCNT-S) via covalent functionalization of MWCNTs with benzenesulfonate groups [69].

Example of covalent functionalization of MWCNTs (functionalization was carried out in water in the presence of sodium sulfanilate and isopentyl nitrite using the Tur reaction) (Figure 5) [69].

Dual functionalization of functionalized carbon nanotubes with amine (CNTs-Amin) and polyhedral oligomeric silsesquioxane (POSS) adsorbed on the PA/EVA composite surface can significantly improve the thermal and mechanical stability of the nanocomposite [70].

Functionalized MWCNTs for PP improve the electrical insulation properties of electrical cables [71]. Obtaining functionalized hydroxyl MWCNTs improves the interaction with the conducting polymer P3HT and increases their diameter to 39.40 from 27.56 nm in the nanocomposite [72].

**Figure 6:**
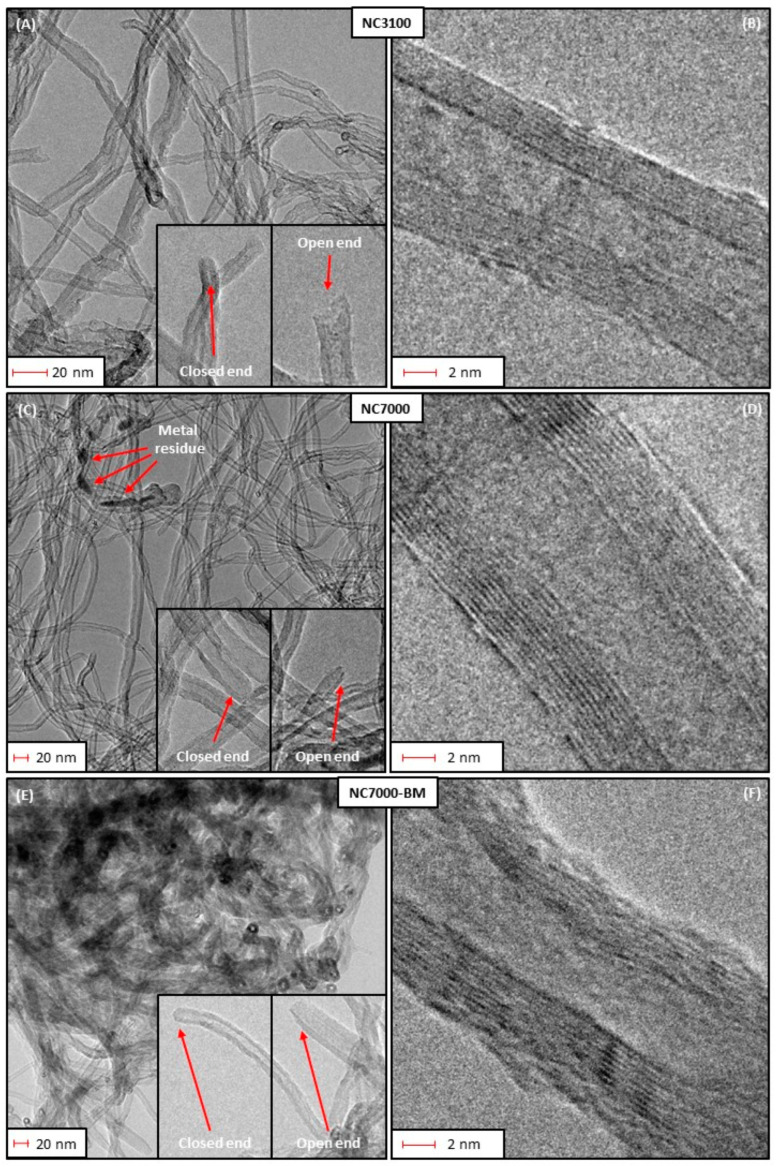
High-resolution transmission electron microscopy (HRTEM) images: (**A**–**D**) obtained CNTs, (**E**,**F**) milled in a ball mill [75].

Treatment of CNTs with strong acid or heating can cause localized structural disruption such as sidewall splitting or unzipping, which requires monitoring of the functionalization process [73]. The mechanisms of interaction at the molecular level between nanotubes and functional groups should be taken into account [74]. To improve the functionalization process, mechano-activation technology, and in particular, the processing of CNTs in a ball mill, can be used, which induces structural changes in the surface (Figure 6) [75].

**Figure 7:**
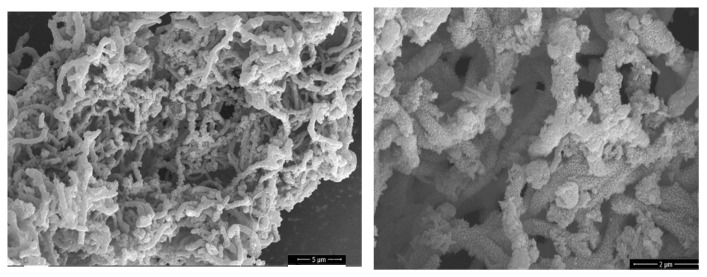
CNTs coated with PANI [77].

One of the directions of functionalization is the use of polyaniline (PANI) [76]. Figure 7 shows the SEM images of PANI-coated CNTs [77].

**Figure 8:**
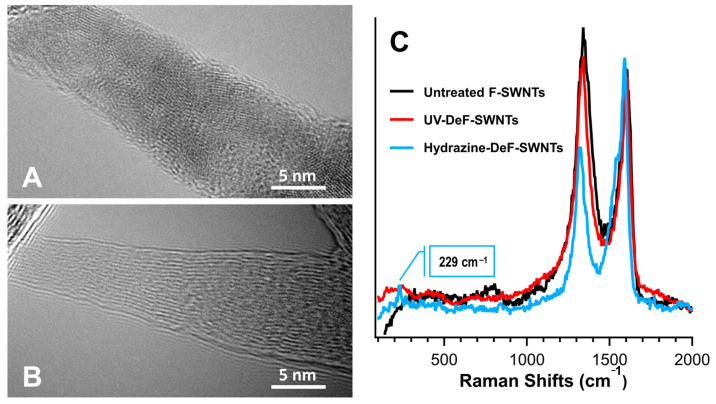
(**A**)—SEM of untreated F-SWNTs, (**B**)—F-SWNTs, (CR) untreated, UV-defluorinated and hydrazine-defluorinated F-SWNTs, (**C**)—Raman spectroscopy [78].

Fluorination can be used to improve the properties of carbon nanotubes. Figure 8A shows F-SWNTs with high surface fluorine functionalization compared to UV-DeF-SWNTs, which have no fluorine on the surface (Figure 8B) [78]. Raman spectra of F-SWNTs and UV-DeF-SWNTs are shown in Figure 8C.

Surface functionalization of carbon nanotubes (CNTs) has become more and more advanced and diverse in recent years. Various functionalization techniques such as wet oxidation (oxidation with nitric acid, sulfuric acid, hydrogen peroxide, potassium permanganate, etc.), dry oxidation (oxidation with air, ozone, plasma, etc.), amidation, silanization, silylation, polymer grafting, polymer wrapping, and fluorination are widely used in practice [79].

## 3. Polymers for Composite Materials

**Figure 9:**
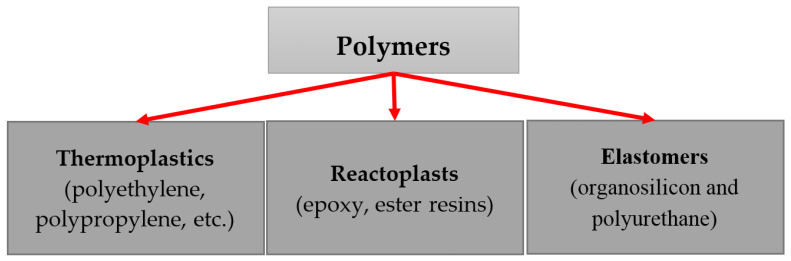
Types of polymers.

Polymers are of great importance in various technological applications; they are used in electronics, medicine, food industry, medicine, energy, construction, etc. Due to their unique physical and chemical properties, polymers have sufficient strength, flexibility, elasticity, and thermal stability, and are chemically inert, etc. Also, today composites based on various types of polymers (Figure 9) can be classified according to their functional purpose, they can be both electrically and thermally conductive, or they combine these two physical properties. PFNs are often used in strain gauge measurements or as materials for electric heating elements.

### 3.1. Epoxy Resins

Various types of nanofillers and polymer additives, such as CNTs, have been used to improve the electrical and thermal conductivity, mechanical properties, flame retardancy, and optical and magnetic properties of EP resins [80,81].

**Figure 10:**
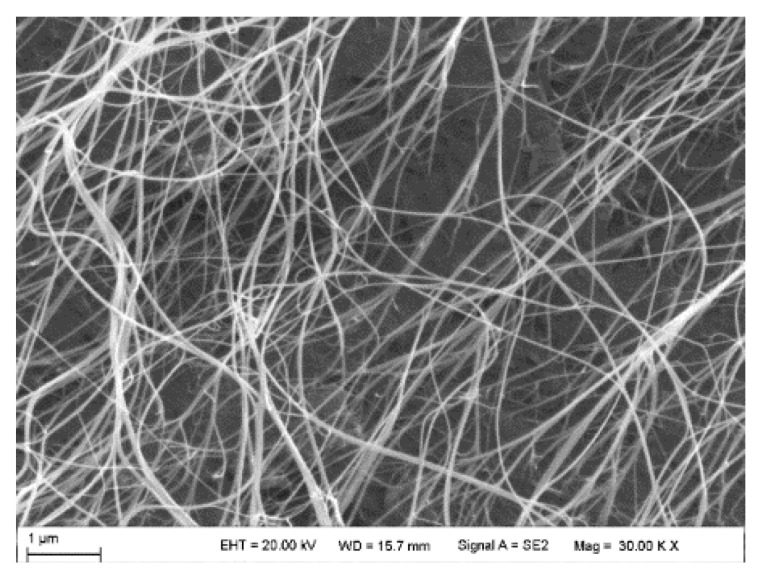
Epoxy resin-based CNT film [85].

A furan bioepoxy monomer (BOMF) cross-linked with isophorondiamine (IPD) and supplemented with CNTs can be used to create electroactive coatings for smart wearable textiles [82]. The addition of a small amount of MWCNTs to epoxy resin improves the mechanical and thermal properties of hybrid nanocomposites [83]. CNT additives in epoxy resins are mainly used to increase their strength [84]. Figure 10 shows the morphological characterization of CNT film based on epoxy resin [85]. Figure 10 shows the carbon nanotube entanglements (cross-overs are formed) in the epoxy resin jetcore.

**Figure 11:**
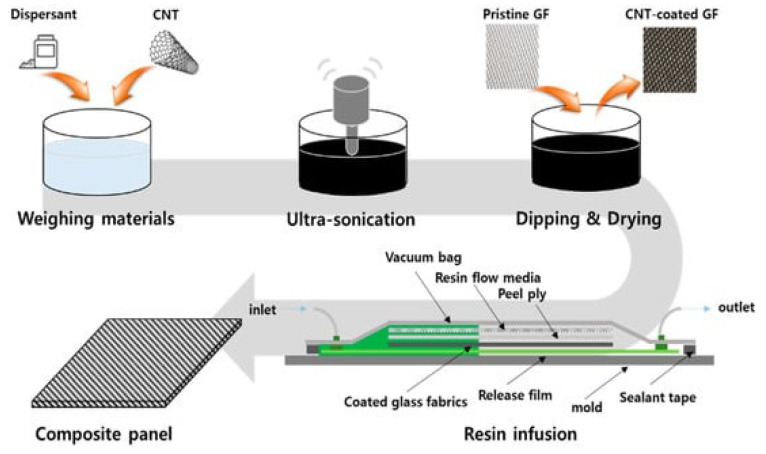
Schematic of the immersion-drying process and resin infusion process for MWCNT-coated composite panel [89].

Technologies used in the production of epoxy resin composites. A significant set of VARTM options for composite manufacturing technologies can be found both in the literature and in industry. Each manufacturing process provides different advantages that must be carefully considered depending on the end application of the desired composite. Vacuum resin transfer molding is possible with different variants of VARTM (DBVI, VAP, and CAPRI), as well as HIPRTM [86]. The vacuum resin transfer molding technology (VARTM) [87] has the disadvantages of void and pore formation as well as variable thickness. There is also double vacuum infusion (DBVI), vacuum-assisted process (VAP), and controlled atmospheric resin infusion (CAPRI) [88]. Figure 11 shows a schematic of the immersion-drying process and resin infusion process for the MWCNT-coated composite panel [89].

**Figure 12:**
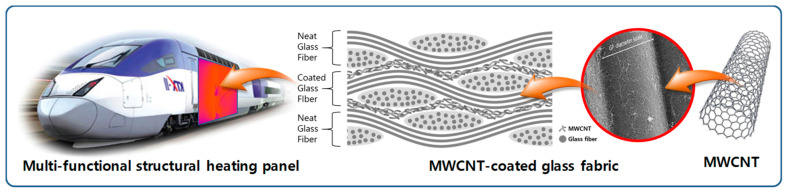
Schematic of MWCNT-coated glass fabric made by resin infusion [89].

A schematic of the MWCNT-coated glass fabric produced by resin infusion is shown in Figure 12 [89].

Vacuum resin transfer molding is widely used in the shipbuilding and wind energy industries for the manufacture of wind turbine blades.

### 3.2. Thermoplastics

**Figure 13:**
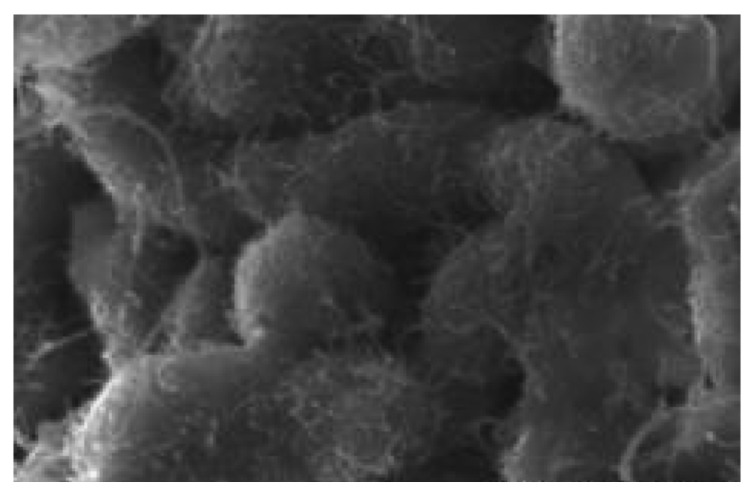
Microphotographs of PESVM with CNTs [94].

The effects of MWCNTs on the thermomechanical and structural properties of high-density polyethylene are presented in [90]. High-density polyethylene (HDPE) has been actively used as thermoplastics to create nanocomposites with CNTs [91]. MWCNTs allow the electrical conductivity of polyethylene (PE) to be improved [92]. The use of fluoroplastic allows a nano-composite containing 20 wt.% of multilayer nanotubes to be obtained [93]. CNTs mixed with the morphology of PESVM with CNTs are shown in Figure 13 [94].

The industrial use of polymers can be grouped into different production methods such as injection molding [95,96,97] and additive manufacturing. Additive manufacturing can be used to recycle thermoplastic materials [98].

### 3.3. Elastomers

**Figure 14:**
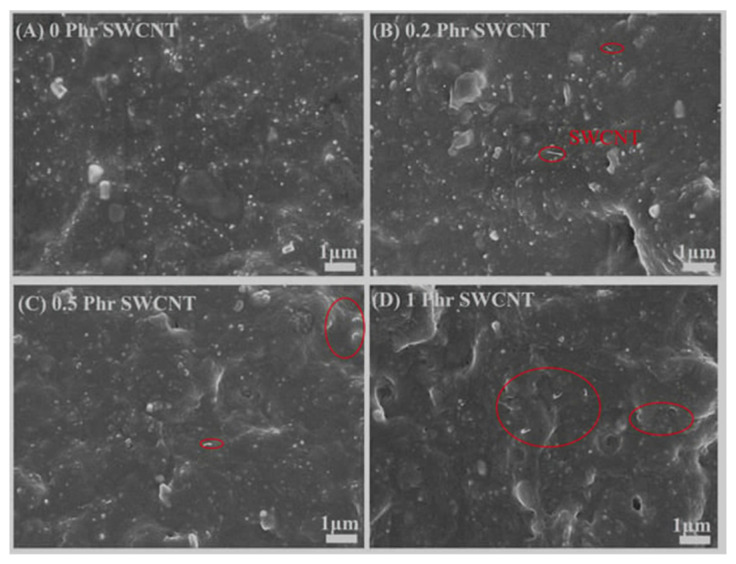
SEM: (**A**) pure NBR/PAE; (**B**) NBR/PAE-0.2SWCNT; (**C**) NBR/PAE-0.5SWCNT; (**D**) NBR/PAE-1SWCNT [101].

In addition to improving mechanical properties, the presence of CNTs significantly improves elastomer properties such as heat resistance and electrical and thermal conductivity [99]. Polyolefin rubber with CNTs can be used for additive manufacturing [100]. In addition, the hardness of NBR/PAE/SWCNT composites gradually increased with increasing SWCNT content (Figure 14) [101]. The morphology of the polymer with CNTs shown in Figure 14 has a compacted appearance, and the CNTs are very tightly enveloped by the polymer matrix. The ovals highlighted in red in Figure 14 refer to SWCNT.

**Figure 15:**
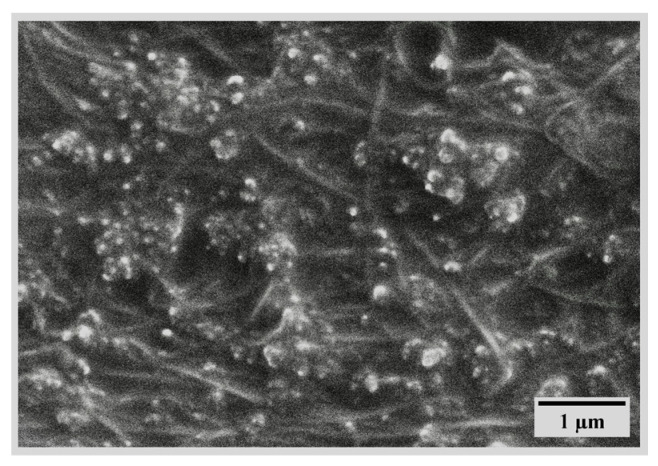
SEM image of the CNT/silicon composite sheet [102].

**Figure 16:**
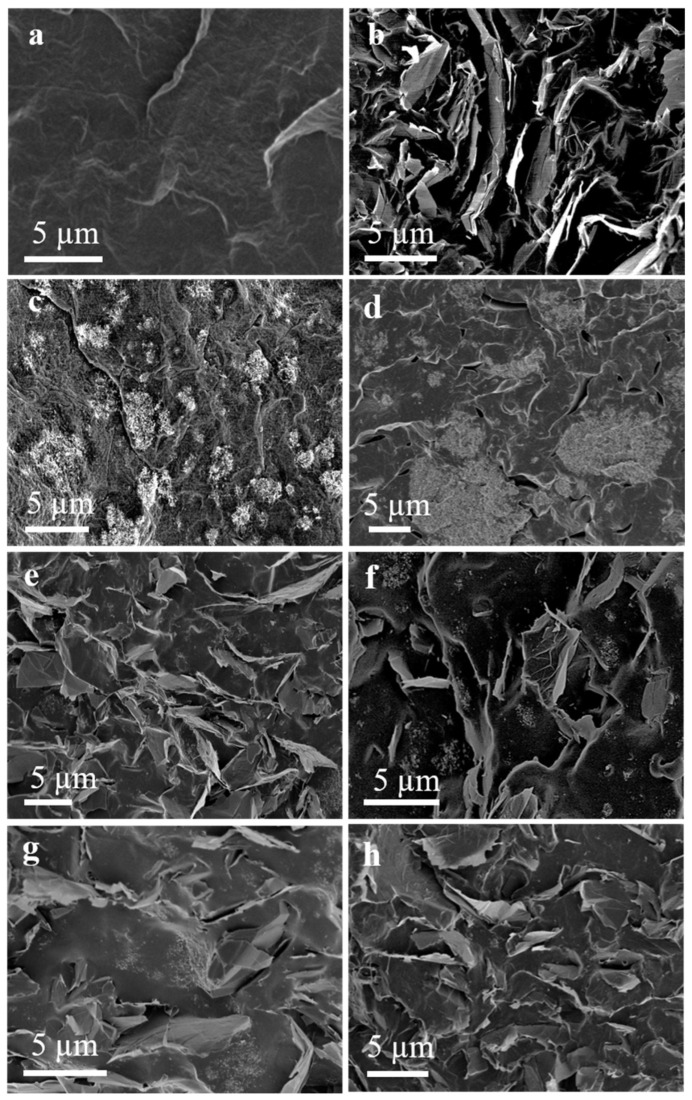
Cross sections of TPU composites obtained by SEM. Sample for DIW formation: (**a**) Pure TPU; (**b**) 10 wt.% GNPs-TPU; (**c**) 10 wt.% MWCNTs-TPU; (**d**) molded shape of 10 wt.% (MWCNTs:GNPS = 1:2)-TPU; Sample for DIW molding: (**e**) 10 wt.% (MWCNTs:GNPs = 3:1)-TPU; (**f**) 10 wt.% (MWCNTs:GNPs = 2:1)-TPU; (**g**) 10 wt.% (MWCNTs:GNPs = 1:1)-TPU; (**h**) 10 wt.% (CNTs:GNPs = 1:2)-TPU [103].

A thin silicone film surrounds the **CNT** filaments, as a result of the sealing step (Figure 15) [102]. CNTs (shown in Figure 16) in the polymer matrix are densely saturated and form developed conductive networks.

The morphology of MWCNT and GNP powder embedded in the TPU matrix is shown in Figure 16 [103]. The combination of MWCNT and GNP forms mutually complementary electrical networks in the composite (Figure 16).

Elastic conducting polymers can be obtained by mixing two polymer components and CNTs. In particular, one of the components may contain a catalyst that promotes polymerization [104]. A rubbing method can be used to produce an elastic layered rubber–graphene composite [105]. Numerical modeling and machine learning can be used to improve the distribution of dispersed fillers in the elastomer matrix structure [106]. Multifunctional polymer nanocomposites containing magnetic nanoparticles (MNPs)—magnetopolymers are presented in [107]. Multicomponent composites based on PLA, PBSA, and PHBV and various copolymers were combined with CNTs, graphene nanoplatelets, and carbon black [108]. The use of polymer additive manufacturing (AM) allows for the creation of an improved material composition and the possibilities of structural modification that AM brings to the synthesis of polymers [109].

## 4. Polymer Conductive Composites

Polymers conducting electric current or possessing any electrical conductivity are actively used in various technical tasks and technologies. There are polymers with their own conductivity [110,111] and polymer composites containing conductive dispersed fillers [112]. Polymer composites are a polymer backbone into which dispersed materials are introduced to promote the charge transfer process [113,114]. Conductive polymer nanocomposites, i.e., polymers reinforced with various nanomaterials, are of particular importance [115,116]. Polymer nanocomposite materials containing carbon nanotubes have a wide demand in various technologies and technical applications, including medical instrumentation: measuring heart rate, heart pulse rate, and temperature [117]. Graphene is also used as dispersed fillers [118]. In [119], the thermal conductivity of polyethylene was found to be improved by using graphene nanoplatelets.

The incorporation of CNTs into polymer matrices significantly improves the conductivity, strength, elasticity, impact strength, and durability of conductive nanocomposites. Formation of a polymer composite containing CNTs typically involves a dispersion process where CNTs reinforce the polymer matrix. The main purpose of dispersion is to uniformly distribute CNTs throughout the polymer so that the composite material has new functional properties. In order to improve the interaction between the polymer and CNTs, the surface of CNTs is modified. There are various methods of CNT surface modification including chemical modification and physical enveloping of CNTs with polymer [120]. In most cases, CNTs have a spider web structure with voids between the nanotube bundles, and such voids can limit the conductivity of the material [121]. It should be noted that metal oxides that interact with CNTs, such as nanoscale iron oxide, can fill the voids and exhibit high activity for energy applications, and the large surface area along CNTs favors their efficient saturation with metal oxide nanoparticles [122]. Moreover, the incorporation of iron oxide nanoparticles reduces the aggregation of CNTs. As a consequence, the functional properties of CNTs are significantly improved after modification with iron oxide.

The combination of dispersed carbon fillers, such as carbon nanotubes (CNTs) and graphene or carbon black [123], can create effective functional properties in a composite. However, mixing bulk materials is a difficult task, so we believe that the co-synthesis of carbon nanomaterials, such as CNTs and graphene, may be the best option to obtain a homogeneous filler structure.

In [124], silver (Ag) and polylactic acid (PLLA) nanoparticles were used in a poly(ε-caprolactone) PLLA/poly(ε-caprolactone)/MWCNTs (PLLA/PCLNT) to form a metalized segregated (IMS) composite.

The electrical, mechanical, piezoresistive, and thermoresistive properties of polysulfone nanocomposites with a total filler concentration of 1 wt.% were investigated using a hybrid combination of multilayer graphene sheets (GLs) and multilayer carbon nanotubes (MWCNTs) in different relative proportions [125]. Nanocomposites consisting of only MWCNTs showed the highest electrical conductivity. On the other hand, the addition of CNTs in the amount of only 25% of the total mass concentration of the filler significantly improved the electrical conductivity of GL nanocomposites. When analyzing the electrophysical parameters of conducting polymer composites, information on percolation thresholds is important. The theoretical model [126] allows us to calculate the percolation threshold for composites with single-walled CNTs and MWCNTs and the electrical conductivity of both the outer metallic and semiconductor shells. Classical percolation theory can be used to describe the percolation threshold.

**Figure 17:**
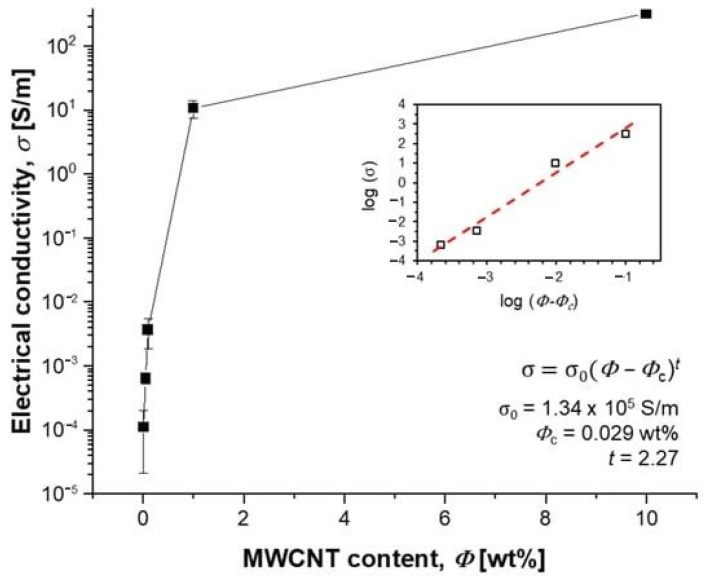
Dependence of conductivity on MWCNT concentration in the composite [127].

σ = σ_0_ (Φ − Φ_c_) t (1)

where σ_0_ is the factor depending on the intrinsic conductivity of MWCNT, Φ is the MWCNT content, Φ_c_ is the percolation threshold, and t is the critical index. By plotting the conductivity according to Equation (1), Φ_c_ of the MWCNT/PDMS composite dispersed in our process was obtained as 0.029 wt.%. In addition, the value of t = 2.27 indicates that a quasi-three-dimensional MWCNT network structure was formed in the PDMS matrix [127] (Figure 17).

Nanocomposites with high conductivity and a strong piezoresistive effect [128] were prepared by adding CNTs coated with the conducting polymer poly(3,4-ethylenedioxythiophene) poly(styrene sulfonate) (PEPS/PSS) to pure insulating polycarbonate. Varying the particle size from 20 to 1200 μm (CNTs /PP) affected the percolation threshold, which varied at concentrations from 1.32 to 0.44 vol% [129]. Sensor polymers form a dynamic class of materials that can change their physical, chemical, or electrical properties (generate a measurement signal) under the influence of temperature, mass, or mechanical changes. Sensing polymers convert these changes into a useful analytical signal [130]. A polymer nanocomposite (PNC) is an enhanced polymer matrix with nanoscale additives [131]. Coordination polymers combine compositional diversity, structural customizability, and technological functionality [132].

CNTs—single-walled CNTs (SWCNTs) and multi-walled CNTs (MWCNTs)—are widely used as dispersed fillers for polymer matrices, which make it possible to obtain functional composites with a variation in mechanical, electrical, and thermal conductive properties. CNTs can be used in the formation of metal–carbon composites [133]. Numerous studies have demonstrated that electrical and thermal conductivity strongly depend on a wide range of parameters: synthesis method; CNTs structure and morphology; polymer type; nanocomposite processing method; dispersion; and agglomeration or alignment of CNTs in the polymer matrix [134,135].

In order to increase the thermal conductivity coefficient (λ) by creating a three-dimensional (3D) structure in polyvinylidene fluoride (PVDF), the technology of electrostatic self-organization of dispersed heterogeneous particles can be used [136]. For this purpose, an electrostatic field is formed between the negatively charged graphene–silver combination (GNs-Ag) and positively charged MWCNTs functionalized with cetyltrimethylammonium bromide (CTAB). The use of SiO/MWCT core-shell hybrid fillers can significantly improve the thermal conductivity [137].

In the work [138], MWCNTs and manganese ferrite nanoparticles (MnFe_2_O_4_) as a magnetic component were used to obtain a composite, which made it possible to increase the absorption efficiency of electromagnetic (EM) waves in the frequency range of 1–41 GHz.

Composite films based on CNTs and para-aramid containing a cyano group exhibit good electrical properties [139]. Hybrid polymer composites exhibit high thermal stability due to the use of polybenzimidazole and MWCNTs [140]. Composite films of sulfonated poly(1,3,4-oxadiazole)/MWCNTs exhibit high thermal stability [141]. Nonlinearity of elec-trophysical characteristics in nanocomposites is related to quantum tunneling [142]. Elec-trically conductive composites (TPU/MWCNTs) can be obtained with tunable mechanical and electrical properties [143]. Table 1 presents a comparison of the effect of different MWCNT concentrations on the electrically conductive properties of nanocomposites based on different polymer matrices.

Polymer nanocomposites serve as the basis for so-called “smart” or “intelligent” materials that have feedback with the environment.

## 5. Polymer Thermally Conductive Composites

Thermally conductive polymers are a class of materials that have the ability to conduct heat, i.e., have a high thermal conductivity coefficient. They are used in various industries: aviation, electronics, medicine, construction, automotive industry, etc. One of the main properties of such polymers is their ability to effectively dissipate heat, which makes it possible to prevent damage and overheating of electronic components.

One of the important areas of development of heat-conducting polymers is to improve their mechanical properties, ensuring durability, as they are significantly reduced due to thermal-oxidative degradation of the material, which determines the reliability of the product as a whole. Polymer heat-conducting composites can be widely used in the production of heat-conducting films, radiators, thermal pads, and other products where high heat transfer efficiency is required. Heat-conducting polymers are widely used in various industries due to the constant development of materials and technologies.

In [153], porous MWCNT foams with a three-dimensional (3D) framework structure were introduced into polydimethylsiloxane (PDMS) to enhance the overall thermal conductivity. The thermal conductivity of 3D MWCNT and PDMS composites can reach 0.82 W/m K, which is approximately 455% higher than that of pure PDMS. Smart nanoparticle technology—thermal conductivity of nanoparticle-filled polymers is shown in [154]. It is shown that thermal conductivity exhibits percolation behavior and is described within the framework of percolation theory. The percolation threshold is 0.6%. It is found that up to the percolation threshold, thermal conductivity correlates well with the degree of crystallinity of the polymer matrix [155]. The molecular chain structure and crosslinking mode of the polymer affect its thermal conductivity [156].

Table 2 shows the thermally conductive nanocomposites based on different types and concentrations of carbon nanostructures and polymer matrices.

The thermal conductivity of nanocomposites varies in a wide range (from 0.859 to 20.55 W·m^−1^·K^−1^) and depends significantly on the type of dispersed nanostructures.

## 6. Polymer Composites for Strain Gauges

Polymers are promising materials in tensometry; this is primarily due to the possibility of their use in composites with specified mechanical properties that are necessary for measuring deformation and stress. The key parameter that determines the possibility of using a polymer for tensometry is resistance to deformation and mechanical strength. A number of polymers show good mechanical characteristics for stretching and compression, in a wide range of temperatures, which, in turn, makes them in demand for use in extreme conditions.

When selecting a polymer for strain measurement, its resistance to fatigue strength and elasticity must also be taken into account. Some polymers have high resistance to long-term loads and allow for stable measurements of strain and stress even under repeated cyclic loads. PFNs have conductive properties after the introduction of CNT-based reinforcing additives.

In addition, PFNs for strain gauges must have good adhesion to ensure reliable fastening and minimize measurement errors. Modified polymers have a functional feature—adhesion with the formation of strong bonds. The choice of polymer for strain gauges depends on specific tasks that determine the range of strain and stress measurements. Thermoresistive response of MWCNT/polymer composites was found for 1 wt% composites at temperatures above −41 °C (232 K), while negative TCRs were experimentally found at temperatures below −41 °C [163]. A composite based on polystyrene/polyurethane and MWCNTs (PS/PU-MWCNTs) with a percolation threshold (about 0.01 wt%) was presented in [164]. The temperature-independent CNTs /epoxy nanocomposite [165].

The conductivity increases linearly with applied compression. This behavior is due to the increase in percolation paths—contacts between adjacent CNTs—under loads [166]. A simple and inexpensive additive method for printing composites based on polyurethane paint that operates efficiently in the temperature range from 0 to 160 °C is presented in [167]. Polyacrylonitrile/CNTs/polyacrylonitrile (PAN/CNT/PAN) can have TCRs in the range of −650 to −900 ppm K^−1^ [168].

The structural properties of the conductive networks affect the strain gauge parameters [169]. Melt mixing technology was used to improve the conductive networks of PVDF/MWCNT composites [170]. The combination of MWCNTs and carbon-coated iron nanoparticles (CCFeNPs) improves the electrical conductivity of the polymer composite [171]. Temperature compensation is realized by four nanocomposite elements, where only two elements are exposed to the measured value [172]. The use of a polymer matrix is of key importance for the formation of a strain gauge [173]. The cohesion of dispersed filler particles depends on the polymer type [174]. Improving the distribution of MWCNTs in nanocomposites reduces the percolation threshold and improves the strain-sensitive properties of MWCNT/PU. The resistance of CNT/polymer strain gauges can be affected by the time factor [175]. The resistance of CNTs is affected by deformation, defects, temperature, chemical effects, and size effects [176].

The combination of carbon fiber (CF), carbon nanotube filaments (CNTs), and carbon nanotube-modified glass fiber (CNTs-GMGF) can have a significant effect on the electrical conductivity of composites [177].

**Figure 18:**
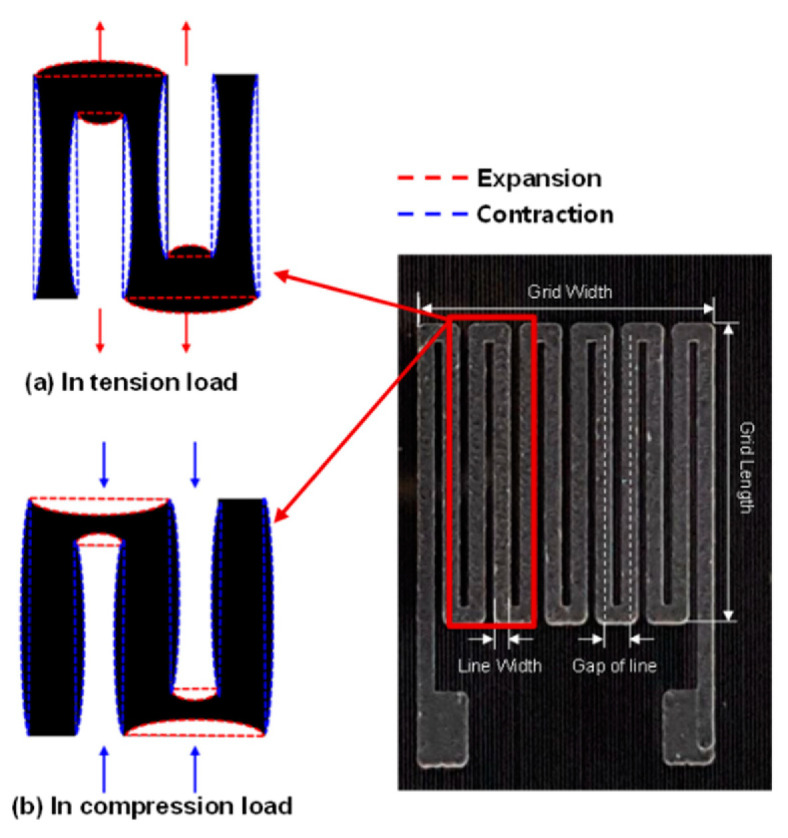
Patterned type of MWCNT/epoxy strain sensor [178].

For MWCNT/polymer-based strain gauges, the shape in which the mechanical loads are measured is important (Figure 18) [178].

It should be noted that strain gauges with MWCNTs can be used to monitor the condition of the structure or detect damage in adhesive joints [179].

## 7. Polymer Composites for Electrical Heating

Polymers for electric heating are widely used in various fields of technology: in the production of heating mats, clothing, medical devices, heated floors, and in industrial processes, etc. Such polymers are becoming increasingly popular due to their operational and technical characteristics: flexibility, high elasticity, anti-corrosion properties, low weight, small dimensions, etc. From a wide range of “smart” polymers, conductive composites that respond to changes in ambient temperature conditions, i.e., possessing the effect of the temperature coefficient of resistance (TCR) should be singled out [180,181]. To obtain conductive polymer composites, dispersed fillers are used, which are used as additives in the dielectric polymer matrix [182]. The high conductivity of the composite requires maximum filler concentration, which leads to a developed internal electrically conductive network [183,184]. However, a high mass concentration of conductive dispersed fillers causes problems associated with poor mechanical characteristics of the composite. At the same time, composites with dispersed fillers show the possibility of their use in conditions of low ambient temperatures [185].

The optimal choice of polymer matrix and conductive dispersed additive achieves the desired mechanical, electrical, and thermal physical characteristics, which, in turn, provides the nanocomposite with various electrical and energy applications, including heat exchange, heat conversion, and materials with a positive temperature coefficient of resistance (PTCR), i.e., electric heating elements with a self-regulating temperature effect [186,187].

To impart improved properties to the composite, different dispersed fillers are combined in various ratios [188,189], which makes it possible to improve both the electrical conductivity and thermal conductivity of the composite during its production [190].

Polymer nanocomposites can be used in electric heating technologies. In order to obtain an electric heater with high elasticity, a polymer elastomer is used. Such heaters are effective in the fight against ice [191]. It should be noted that for individually used CNTs there is a disadvantage in the form of agglomeration, and for graphite, excessive compaction is characteristic, which leads to uneven distribution in the volume of the composite.

One of the functional features of conductive polymer composites is their ability to transition between insulating and conducting states as a result of a change in their intrinsic resistance, which is correlated with temperature [192,193]. Phase processes in a conducting composite can be affected by electron beam pre-irradiation [194] as well as by the structural properties of the filler [195]. These composites are characterized by a noticeable increase in resistance with increasing temperature, especially near the melting temperature (Tm) of the crystalline polymer, a phenomenon known as the positive temperature coefficient of resistance (PTCR) effect [196,197,198].

To improve composites with PTCR, it is necessary to reduce electrical resistance at room temperature and increase resistance near the polymer melt temperature [199]. In this regard, it is necessary to optimize the concentration of dispersed filler and analyze the interaction of dispersed particles with the polymer matrix. Modification of polymers with carbon nanostructures obtains new functional materials with improved performance characteristics, which include electrical and thermal parameters.

In [200], 0D silver-coated glass spheres, 1D CNTs, and 2D graphene nanoplatelets were used as typical conductive fillers, and high-density polyethylene, thermoplastic polyurethane, and polycarbonate were used as polymers. The highest intensity of the PTCR was observed near the “critical” percolation threshold, which corresponds to conductive networks with the smallest number of interparticle contacts.

The CNT-based heater [201] achieves a maximum temperature of 95.0 °C, while the AgNP/CNT hybrid heater achieves a higher maximum temperature of 118.6 °C at 8 V. Heaters based on MWCNT films can achieve a large area (more than 30 × 30 cm^2^) [202].

Reduced graphene oxide (rGO)/carbon nanotubes/natural rubber (GO/CNTs/NR) composites are heated to 69.1 °C at an input voltage of 15 V [203]. Optimization of the structural properties of the polymer and conductive filler can significantly improve the uniformity of the temperature field distribution on the surface of the PTC heater [204]. The use of graphene nanoplates and CNTs allows the use of an electric heating mode with a voltage of 200 V and a temperature of 125 °C [205]. A copolyester thermoplastic elastomer can provide the switching temperature of PTC using electrical heating at 200 °C (voltage 240 V) [206]. A combination of a water-based polyurethane (WPU) matrix and CNTs and calcein-modified graphene (CAG) nanosheets allows for heat dissipation at 5 V [207].

**Figure 19:**
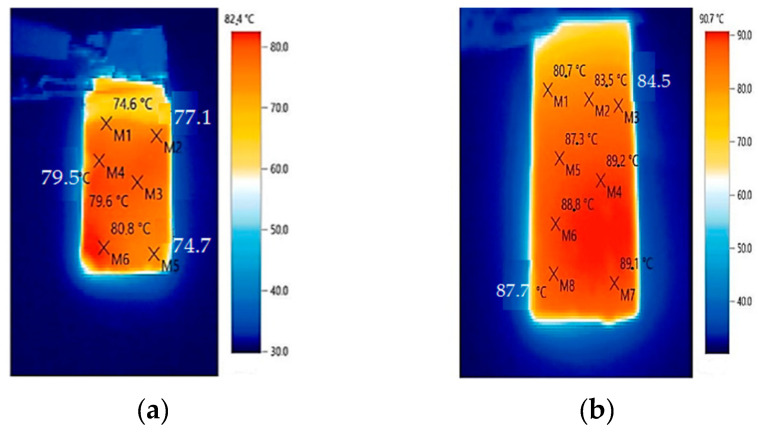
Thermograms: (**a**)—elastomer with MWCNTs and Ni; (**b**)—elastomer with MWCNTs and Cu [208].

The efficient operation of an elastic heater with a voltage of 220 V at a temperature of 90.7 °C is presented in Figure 19 [208].

Table 3 presents heaters based on composites with polymer matrices and dispersed fillers, operating at various electrical voltages and temperature conditions.

**Figure 20:**
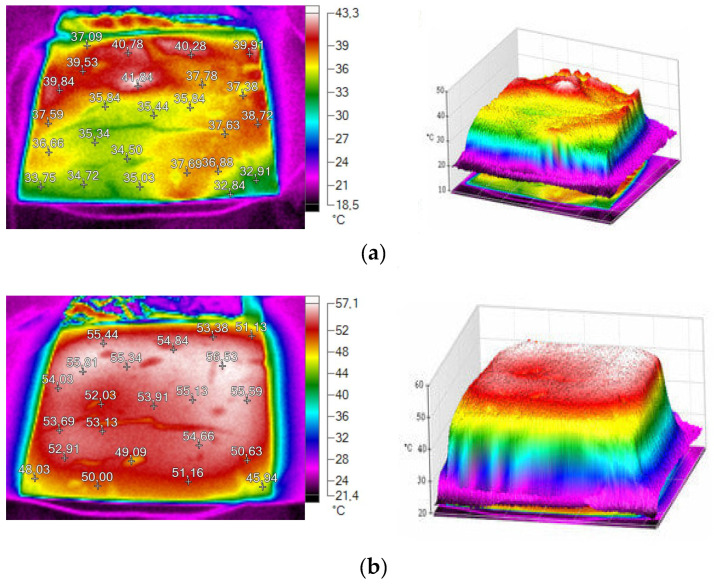
Thermograms: (**a**) elastomer with MWCNTs and graphite: the 1st stage of mechanical activation (mixing); (**b**) elastomer with MWCNTs with graphite: the 2nd stage of mechanical activation [213].

Improvement in the uniformity of the temperature field distribution on the surface of the heater can also be achieved by using mechanical activation of MWCNTs, which helps to reduce the agglomeration of nanosized particles (Figure 20) [213].

The main advantage of polymer composites with PTCR for electric heating is energy savings. They significantly reduce heating costs due to the effect of self-regulating temperatures. Polymer composites have a fast heating time, and high thermal conductivity, as well as plasticity and corrosion resistance.

Nanomaterials surpass classic carbon black and soot in potential capabilities related to electrical and thermal conductivity and achieve good results at significantly lower concentrations. The higher cost of nanomaterials is compensated for by improved electrophysical characteristics at significantly lower concentrations. Comparisons of nanocomposites with CNTs and GNPs, as well as a combination of GO/CNTs in the heat release mode, were carried out. Studies show the possibility of comparable results in electrical and thermal characteristics, but CNTs are better suited for the formation of PTCR in a nanocomposite, since they allow the effect of tunnel conductivity to be realized and are cheaper than graphene. CNTs are also mass-produced, unlike other types of nanomaterials.

## 8. Conclusions

Carbon nanomaterials (CNM) have high electrical and thermal conductivity, mainly belonging to the class of thermoresistive materials, i.e., electrically conductive, which exhibit a thermoresistive effect—a phenomenon consisting of a change in specific electrical resistance under the influence of temperature. A rational option for creating a thermoresistive effect in polymer composites is carbon nanotubes (CNTs), due to their high bulk density and best affinity with the polymer matrix compared to other CNMs.

The use of CNM in polymer matrices allows for the formation of a general class of composites—heat- and electrically conductive polymer composites. Various combinations of polymers and dispersed fillers affect the formation of a positive temperature coefficient of resistance, which allows the effect of temperature self-regulation to be realized.

The methods of CNT synthesis are considered, providing a large morphological and structural diversity of dispersed nanomaterials for various technical applications. The following requirements are imposed on conducting dispersed fillers:

- Dispersed conductive fillers can be either metallic or carbon. Depending on the affinity with the polymer;
- CNTs mold into PTCR at lower concentrations, which maintains mechanical strength and uses more efficient heat transfer modes;

The use of thermoplastic elastomers ensures the switching temperature of PTCR during electric heating at a level of 200 °C (voltage 240 V).

CNTs create strain gauge transducers with greater sensitivity and a wide measurement range.

- Comparisons of nanocomposites with CNTs and GNPs, as well as a combination of GO/CNTs in the heat release mode, were conducted. Our studies show the possibility of comparable results in electrical and thermal characteristics, but CNTs are better suited for forming PTCR in a nanocomposite, since they allow the effect of tunnel conductivity to be realized.

The advantage of polymer composites with PTCR for electric heating is high energy efficiency. They significantly reduce the costs of electric heating due to the effect of self-regulation of temperature (resistive properties).

## Figures and Tables

**Table 1:** Electrically Conductive Composites (Polymer/CNT).

№	Nanocomposites	Mass and Volume Concentration of Carbon Nanotubes	Electrical Properties	Literary Source
1	Phenolic resin/MWCNT	0.210 wt.%	47.5 S/m	[89]
2	MWCNT/ABS	9.09 mas. %	0.15 Ω m	[142]
3	polyurethane/MWCNT MWCNT- COOH	1 mas. %	10^−5^ S cm^−1^	[144]
	MWCNT-NH_2_		7 S cm^−1^	
4	CNTs/polyamide	2 mas. %	1.5 × 10^6^ Ω/sq	[145]
5	poly(acrylonitrile)/SCNT	40 wt. %	1.5 × 10^4^ S m^−1^	[146]
6	Polyaniline/MWCNT	80 wt. %	2.5 × 10^3^ S m^−1^	[147]
7	MWCNT/ABS	8 wt. %	1.8 Ω cm	[148]
8	CNTs/ABS	10 mas. %	0.65 Ω cm	[149]
9	MWCNT/epoxy resin	4 mas. %	6 × 10^−2^ S/cm	[150]
10	MWCNT/ABS	10 mas. %	10^−5^ S/cm	[151]
11	SWCNT/polycarbonate	3 wt %	10^−7^ С/cm	[152]

**Table 2:** Thermal properties of CNT/polymer composites reported by different researchers.

№	Materials	Concentration	Thermal Conductivity, W·m^−1^·K^−1^	Reference
1	MWCNT/PP	79 vol.%	0.859	[157]
2	s-CNTs/PU	1 mas.%	4.3	[158]
3	s-CNTs/PA12	1 mas.%	16.9	[158]
4	SWNT/PVDF	49 vol.%	1	[159]
5	EPR composites with hybrid core-shell fillers SiO/MWCT	1 mas.%	0.55	[137]
6	Epoxy/CFs/CNTs	17.60 vol.% CFs and 8.42 vol.% CNTs	20.55	[160]
7	EVA/MWCNT	20 wt.%	2.318	[161]
8	CNTs/polybenzimidazole polymer nanofiber composites	1.94 wt.%	18	[162]

**Table 3:** Comparison of different polymer nanocomposite heaters.

Heater	Voltage/Temperature, [V]/[°C]	Reference
AgNP/CNTs	8/118.6	[201]
Heaters based on films with MWCNTs	12/90	[202]
GO/CNTs/NR	15/69.1	[203]
Multi-directional CNTs	12/100	[204]
CNTs/GNP/HDPE	200/125	[205]
TPE/GNP	240/200	[206]
CAGn-CNTs/WPU	5/72	[207]
Elastomers/MWCNTs	220/90.7	[208]
poly(m-phenylene isophthalamide) (PMIA)/carbon black (CB)	20/200	[209]
polyimide (PI)/carbon black (CB)	60/270	[210]
G-CNTs/TPU	140/94.05	[211]
CNTs/PDMS	5/104	[212]
CNTs/Nylon 6 c	5/102.5	

## Data Availability

The data presented in this study are available on request from the first author.
